# Results from an Online Computer-Tailored Weight Management Intervention for Overweight Adults: Randomized Controlled Trial

**DOI:** 10.2196/jmir.1901

**Published:** 2012-03-14

**Authors:** Lenneke van Genugten, Pepijn van Empelen, Brigitte Boon, Gerard Borsboom, Tommy Visscher, Anke Oenema

**Affiliations:** ^1^Department of Public HealthErasmus MCUnivserity Medical CenterRotterdamNetherlands; ^2^TNO Quality of LifeLeidenNetherlands; ^3^Trimbos InstituteNetherlands Institute of Mental Health and AddictionUtrechtNetherlands; ^4^Research Centre for Overweight PreventionZwolleNetherlands; ^5^Department of Health Education and Health PromotionCare And Public Health Research Institute (CAPHRI)Maastricht UniversityMaastrichtNetherlands

**Keywords:** Prevention, Overweight, Adults, Randomized Controlled Trial, Physical activity, Dietary intake, BMI

## Abstract

**Background:**

Prevention of weight gain has been suggested as an important strategy in the prevention of obesity and people who are overweight are a specifically important group to target. Currently there is a lack of weight gain prevention interventions that can reach large numbers of people. Therefore, we developed an Internet-delivered, computer-tailored weight management intervention for overweight adults. The focus of the intervention was on making small (100 kcal per day), but sustained changes in dietary intake (DI) or physical activity (PA) behaviors in order to maintain current weight or achieve modest weight loss. Self-regulation theory was used as the basis of the intervention.

**Objective:**

This study aims to evaluate the efficacy of the computer-tailored intervention in weight-related anthropometric measures (Body Mass Index, skin folds and waist circumference) and energy balance-related behaviors (physical activity; intake of fat, snacks and sweetened drinks) in a randomized controlled trial.

**Methods:**

The tailored intervention (TI) was compared to a generic information website (GI). Participants were 539 overweight adults (mean age 47.8 years, mean Body Mass Index (BMI) 28.04, 30.9% male, 10.7% low educated) who where recruited among the general population and among employees from large companies by means of advertisements and flyers. Anthropometric measurements were measured by trained research assistants at baseline and 6-months post-intervention. DI and PA behaviors were assessed at baseline, 1-month and 6-month post-intervention, using self-reported questionnaires.

**Results:**

Repeated measurement analyses showed that BMI remained stable over time and that there were no statistically significant differences between the study groups (BMI: TI=28.09, GI=27.61, *P*=.09). Similar results were found for waist circumference and skin fold thickness. Amount of physical activity increased and intake of fat, snacks and sweetened drinks decreased during the course of the study, but there were no differences between the study groups (eg, fat intake: TI=15.4, GI=15.9, *P*=.74). The first module of the tailored intervention was visited by almost all participants, but only 15% completed all four modules of the tailored intervention, while 46% completed the three modules of the general information intervention. The tailored intervention was considered more personally relevant (TI=3.20, GI=2.83, *P*=.001), containing more new information (TI=3.11, GI=2.73, *P*=.003) and having longer texts (TI=3.20, GI=3.07, *P*=.01), while there were no group differences on other process measures such as attractiveness and comprehensibility of the information (eg, attractive design: TI=3.22, GI=3.16, *P*=.58).

**Conclusions:**

The online, computer-tailored weight management intervention resulted in changes in the desired direction, such as stabilization of weight and improvements in dietary intake, but the intervention was not more effective in preventing weight gain or modifying dietary and physical activity behaviors than generic information. A possible reason for the absence of intervention effects is sub-optimal use of the intervention and the self-regulation components. Further research is therefore needed to gain more insight into how the intervention and exposure to its contents can be improved.

**Trial Registration:**

NTR1862; http://apps.who.int/trialsearch/trial.aspx?trialid=NTR1862

## Introduction

Obesity (Body Mass Index: BMI > 30 kg/m^2^) is a major public health concern, because of its high prevalence and association with several negative health outcomes [1-3], a lower quality of life [4,5], and increased health care costs [6]. Given the poor long-term results of weight loss attempts among obese people [7], prevention of weight gain has been postulated as an important strategy for fighting the obesity epidemic [8]. Prevention of weight gain is particularly important among people who are overweight (BMI 25-30 kg/m^2^), since they are most at risk of becoming obese. Weight gain prevention (WGP) or modest weight loss does not require drastic dieting but can be achieved by making small, sustained changes in dietary intake or physical activity. In the Netherlands, the average annual weight gain was about 0.5 kg [9]. The global average 1 kg of annual weight gain that is seen in many populations is caused by excess energy intake of about 7000 kcal a year. In reverse, it is hypothesized that 1 kg of annual weight gain can be prevented in about 90% of the population by a daily decrease in dietary intake (DI) or increase in physical activity (PA) of about 100 kcal [10]. Currently, there are only a few effective interventions that focus on the prevention of obesity in overweight adults [11] that take the small changes approach and that can reach large numbers of people. Therefore, we developed a computer-tailored intervention aimed at preventing weight gain among overweight adults [12] and evaluated it for effects.

### Behavior Change and Online Interventions

Evidence of the effectiveness of current obesity prevention interventions indicates that such interventions can be successful, but that findings are mixed. In the *Pound of Prevention Study*, a monthly newsletter (including practical guides for behavior change, recipes and locations for walking) and other activities such as weight control sessions were found to have favorable (but statistically non-significant) effects on behavior and weight [13]. Using 10 modules of usual care and email counseling [14] or individual and environmental intervention strategies [15], 2 Dutch worksite interventions were successful in changing behavior and/or preventing weight gain. In a review by Lemmens et al [16], 4 of 11 studies reported a positive effect on weight, of which 3 were aimed at modifying DI and PA; more intensive and sustained interventions, including monitoring of behavior, were more effective [16]. Kremers et al [17], in their meta-analysis on prevention of overweight and obesity, reported a small, but statistically significant average effect size (d=0.06) of weight management interventions, with studies aiming at weight management being more successful than studies aiming at behavior change for other reasons (eg, decrease risk for coronary event). However, most of these studies were not aimed at overweight adults [17].

One key factor in successful WGP is that changes in DI and PA need to be maintained for a long time, which requires self-regulation skills. Self-regulation [18,19] motivates and enables people to achieve self-set goals. The first step in self-regulation consists of setting a goal, in this case WGP. Second, one has to choose the means to achieve this goal. In the case of WGP this means deciding to make small changes in DI and/or PA. Third, a person needs to make a detailed plan for how to make the desired change and how to avoid difficulties that may occur when making the desired change. The step of planning is followed by actual goal pursuit, monitoring, and evaluation of progress toward goal achievement. Providing a platform for goal setting, planning, monitoring, and providing feedback on targeted behavior has been identified as pre-requisite for interventions that aim for change in dietary intake and physical activity [20]. Another prerequisite for WGP interventions is that large numbers of people need to be reached, with relative low costs per person, since about 35% of the adults in the Netherlands are overweight [21].

To be able to meet the criteria of an individualized approach and large reach, we chose to develop an online, computer-tailored intervention. Several reviews have shown that (online) computer-tailored interventions may have a positive effect on energy balance-related behaviors [22-25] compared with general information or no information. One review included 76 studies on eating a healthy diet and/or undertaking physical activity and found a statistically significant overall small effect size (g=0.17) for computer-tailored interventions [22]. Kroeze et al concluded in a review that there is consistent evidence that tailored interventions have a positive effect on dietary intake, especially on fat reduction, and probably on physical activity [23]. Of the 10 studies that were included in the review of Neville [25] and that were aimed at reduction of dietary fat intake, 5 found a positive effect of ‘second generation’ (delivered through interactive technology or desktop applications such as websites, email and CD-rom) tailored interventions. A review on physical activity showed that 10 of 16 studies found statistically significant positive effects on physical activity and weight reduction measures [24]. These reviews show that positive effects on weight, dietary and physical activity behaviors can be achieved with computer-tailored interventions, but there is currently no evidence for the efficacy of a tailored intervention on prevention of weight gain among overweight adults.

### Aims of the Study

The aim of this study was to establish the efficacy of an online, computer-tailored weight management intervention (GRIPP) on anthropometric outcome measures at 6-months post-intervention and on energy balance-related behaviors (intake of sugar-sweetened drinks, snacks and fat, and physical activity) at 1- and 6-months post-intervention compared to a generic information control group. The hypotheses were that anthropometric outcomes (BMI, waist circumference, and skin fold thickness) will be more favorable at 6-months post-intervention for the intervention group, because the average annual weight gain will occur in the control group and not in the intervention group, and that the intervention group will have lowered intake of sugar-sweetened drinks, snacks and high fat products, and engage in more PA at 1- and 6-months post-intervention, as compared with the control group.

In addition, we performed a process evaluation in order to contribute to a better understanding of the (non) effects of the intervention and to identification of areas for improvement for the intervention [26]. Therefore, measures of use and appreciation were included in the process evaluation.

## Methods

### Study Design

A 2-group randomized controlled trial was conducted in which the computer-tailored intervention (TI, n = 270) was compared with a generic information intervention (GI, n = 270) control group. Body height, weight, waist circumference, and skin fold thickness were measured at baseline and 6-month post-intervention and measurements of energy balance- related behavior were taken at baseline, 1-month post-intervention and 6-month post-intervention. After baseline assessment, participants were allocated to one of the two study groups (1:1) by means of sex-stratified computer block randomization (block size: 10). The study received a declaration of no objection from the Erasmus MC Medical Ethics Committee.

**Participants**

The participants were adults (18-65 years) who were overweight (self-reported BMI 25-30 m^2^). Exclusion criteria were not having a sufficient command of the Dutch language, not having Internet access, being pregnant, following a diet prescribed by a physician or dietician, and having a history of depression or eating disorders.

A power calculation showed that 200 participants in each study group would be sufficient to detect 0.4 difference in BMI points between the intervention and control group (caused by weight gain of 0.3 kg in the control group and weight maintenance or slight weight reduction in the intervention group) with a power of 0.80 and a significance level of *P*<.05. To account for dropout between the measurements, 600 participants needed to be recruited for the study.

Participants were recruited between March and October 2009 from the general population in the Rotterdam (the second largest city in the Netherlands, with approximately 600,000 inhabitants) region through advertisements in local newspapers, flyers that were delivered door-to-door, in waiting rooms of GP’s, and among the employees of 4 large companies, with the aim to reach a diverse population with respect to socioeconomic status. The recruitment materials contained information about the goal, process and incentives for the study. More detailed information was available on the study website. People who were interested in participating in the study were asked to fill out an online subscription form available on the study website that was used to assess whether they were eligible for participation in the study. Participants were included in the study if their BMI (calculated as weight/height^2^), based on self-reported height and weight, was between 24 and 31 (this range was broader than the objective inclusion criteria of a BMI of 25-30, in order to prevent exclusion based on biased self-reported measures). If people did not meet the inclusion criteria, they could not subscribe for the study.

### Procedures

After subscription, participants received a confirmation letter and information leaflet about the study. In addition, they received an email in which they were asked to fill out the baseline questionnaire online. Weight, height, waist circumference and skin folds were measured at the hospital site. Participants signed the consent form when they had their anthropometric measurements taken. When participants did not come to have their anthropometrics measured, the consent form was sent to their home address with a return-envelope. Participants preferably completed both measurements (anthropometrics and questionnaire) but were also randomized when they had completed only one measurement.

All randomized participants received a login name and a password by email, which gave them access to the allocated intervention program. Participants were asked to visit the websites at least three or four times during a 2-month period. They received email reminders to (re-) visit the intervention every 2 weeks. At 1 month and 6 months after the intervention period, participants were asked by email to fill out the online questionnaire again. Furthermore, after 6 months they were again invited to the hospital site for measurement of weight, waist circumference and skin fold thickness. Participants who did not respond to the email invitations for the anthropometric measurements or to complete the questionnaire received a phone call to motivate them to complete the questionnaire or to have their anthropometric measures taken. Gift vouchers were handed out as compensation for travel expenses and invested time. Participants who filled out the questionnaire 1-month post-intervention received a gift voucher of € 10. Participants who filled out the questionnaire and had their anthropometrics measured at 6-months post-intervention, received (another) gift voucher of € 10. Furthermore, because dropout at 1-month post-intervention was higher than the expected 10%, 10 extra gift vouchers of € 20 were raffled among the participants who completed all measures at 6-months post-intervention.

### Outcomes/Measures

#### Anthropometric Measures

The body measurements were performed by trained research assistants following a measurement protocol. *Body height* was measured twice using a Seca mobile height rod with an accuracy of 0.1 cm. The mean of both measures was used for height. A calibrated electronic digital floor scale (Seca 888 clas III) was used to measure *body weight*, with an accuracy of 0.2 kg. The measures of height and weight were used to calculate *BMI* (weight [kg] / (height [m])^2^). *Waist circumference* was measured twice with a flexible band (Seca 201) with a precision of 0.1 cm. When the difference between two measurements was larger than 1.0 cm, the waist circumference was measured twice again. Mean waist circumference was calculated based on the last two measurements. *Skin fold thickness* was measured at 4 sites (biceps, triceps, subscapular and supra-iliac) with the Harpenden Skinfold Caliper to assess body fat percentage [15,27]. Each site was measured 3 times and the mean was calculated for each site. A variable for total skin fold thickness in centimeters was composed by summing the means for the 4 sites in 1 measure. The same measures, except for height, were taken at baseline and 6-months post intervention.

#### Energy Balance-Related Behaviors

In this study, we examined the effects of the interventions on 1) fat intake, 2) snack intake, 3) intake of sweetened drinks (mean number of sweet and sweetened drinks per day), and 4) physical activity. *Fat intake,* expressed as ‘fat score’, was assessed using a food frequency questionnaire assessing the frequency and quantity of a variety of high density foods eaten in the past week. It was based on a validated questionnaire [28] and allows for calculating fat intake and intake from specific food groups. The questionnaire consisted of 74 questions and was organized according to meal pattern. Participants recorded their frequency of consumption and portion size for a selection of food items eaten during meals or between meals. Fat points were based on frequency and amount of intake of high fat products, with higher scores indicating higher fat intake. There were 23 products, in the following categories: dairy products (5), butter (1), gravy (1), sandwich fillings (3), meat and cheese for main dinner (2) and snacks (sweet, salty, hot and cold; 11 in total). There were a maximum number of points (2 to 5) for each product. In total, a maximum of 83 fat points could be obtained.

Furthermore, people were asked to answer questions about the mean number and amount of sweet and salty, and hot and cold snacks per week from 11 categories (eg, fried products, candy bars, etc). *High energy snack intake* was calculated as the mean number of high energy snacks per day by multiplying the frequency per week with the amount per frequency, divided by 7 (days a week). To assess intake of sweetened drinks, questions on frequency and amount for fruit juices, soft drinks and sweetened tea and coffee were asked. *Intake of sweetened drinks* was calculated in a similar way as intake of snacks.

*Physical activity* was assessed using a questionnaire based on ‘The Short Questionnaire to Assess Health - Enhancing Physical Activity’ (SQUASH), developed to assess habitual activity level [29]. In this 16-item questionnaire, participants were asked to indicate on how many days of the week they participated in specified activities, and how much time they engaged in the activity per occasion. For active transport, respondents were asked how often they cycled and walked for home to work transportation, and the duration. Similar questions were asked for walking and cycling during leisure time. Furthermore, participants were asked how many different sports they did on a weekly basis (with a maximum of 4). For each different sport, they were asked to choose the type of sport (eg, swimming, yoga, running) from a list, and to choose the weekly frequency, and the average duration per activity. For each category, the average number of minutes per week was calculated by multiplying the frequency with the duration. Then, this number was divided by 7 to calculate the mean number of minutes per day. Next, the number of minutes engaged in physical activity per day was calculated as the sum of all activities (active transportation, leisure time activities, and sports). The same questionnaires were used at baseline, 1-month and 6-month post-intervention to assess energy balance related behaviors.

### Demographic Factors

*Sex* (male/female), date of birth, and educational level were assessed in the baseline questionnaire. To determine a*ge* we asked for date of birth. *Education* was assessed by asking the participants to indicate their highest completed education (8 answering options). A 3-category variable was subsequently made, indicating a low (completed no education, primary school, secondary school or lowest level of high school or lower vocational training), medium (intermediate or high level high school) or high (completed higher vocational training, college or university) level of *education*.

### Process Measures

An *objective measure of exposure* to the intervention was obtained from the login data from the intervention registration, which keeps information from participants’ use of the (tailored) information. One scale was made for use/exposure, indicating the number of modules actually used (0-4 for TI, 0-3 for GI). Self-reported measures of use and appreciation were included in the 1-month post-intervention questionnaire. If not stated otherwise, answer categories ranged from ‘totally disagree’ (1) to ‘totally agree’ (5).

*Amount of information read* was assessed by the question, “To what extent did you read the information in the program?” Answering categories ranged from ‘none of it’ (0) to ‘all of it’ (5). *Perceived length of the text* was assessed by the question, “What do you think of the lengths of the text in the program?” Answering categories ranged from ‘much too short’ (1) to ‘much too long’ (5). Perceived *personalization* was assessed with the statement, “The information in the program was relevant for me personally”. *Novelty* of the information was assessed by, “The information in the program was new for me”. *Usefulness* of the information was assessed with, “The information in the program was useful”. *Attractiveness* was assessed by the question, “The design of the program is attractive”. *Usefulness of the intervention* was assessed with the question, “the program is a good instrument to control my weight”. Furthermore, participants were asked whether they would *recommend the intervention to others* using the response options, ‘no’ (1), ‘maybe’, (2), or ‘yes’ (3). Participants were also asked to give an *overall grade* to the intervention on a scale from 1 to 10 (1 being very low, 10 being very high). These questions were included in the questionnaire for the intervention and the control group and are only reported for those who have actually used the interventions.

### The Intervention

The objective of the computer-tailored intervention was to prevent weight gain in adults who are overweight by inducing small changes (100 kcal/day) in energy balance-related behaviors (DI and PA). It aimed at making a change in (one or more) behaviors that add most to the energy balance and that are associated with weight gain (frequency and duration of various physical activities and intake of fat (from several categories such as dairy, meat, cheese, sauce, snacks and sweetened drinks) [30]. The intervention was carefully developed based on theory and evidence using the Intervention Mapping approach [26]. The intervention goals, methods, and strategies were based on self-regulation theory [19], and other theories such as the Theory of Planned Behavior [31], Precaution Adoption Process Model [32] and implementation intentions [33]. The strategies were combined into a computer-tailored Internet-delivered intervention. Detailed information about the intervention development and content is described elsewhere [12].

The intervention consisted of 4 modules, each to be visited 1 week after the previous one and followed the steps of self-regulation. Completion of the entire program took about 90 minutes in total. The first module aimed at commitment to prevent weight gain by considering pros and cons of WGP, identifying and setting a goal for one relevant change in DI or PA and making a plan for change. Participants were made aware of current levels of DI and PA and possibilities for change by providing them with individualized feedback on their behavior. Then, people could make a choice for what to change (guided goal setting) and make a plan for where, when, and how to make that change (implementation plan). The second and third modules were focused around evaluation of progress toward behavior change, and provided feedback on past week performance. If necessary, it supported adaptation of action and coping plans (when attempts to change behavior were unsuccessful). The fourth module was aimed at promoting sustained self-regulation of body weight without use of the program. A tool to monitor and evaluate (changes in) body weight was provided, as well as a short guideline with sequences of actions for long term WGP, reflecting on the self-regulatory skills that had been practiced in the previous three modules, and provision of positive reinforcement to maintain behavior. At the end, the participants signed a personalized contract, which included their personal behavior goals, actions plans, weight status, etc. The tailored modules were embedded in a website that also contained recipes, a peer-to-peer forum and links to useful websites, and was accessible through the Internet.

The generic information for the control group was embedded in a website with similar content, and similar reminders were sent to the participants. The main components of this website were 3 modules with general information on weight gain prevention, which had a similar layout as the TI. The first module aimed at increasing the motivation for WGP. The second module aimed at choosing a behavior change by providing information about possible changes. The third module provided general information about a healthy diet and safe physical activity.

### Statistical Analyses

Descriptive statistics were used to characterize both study groups at baseline. Logistic regression analyses were conducted to evaluate whether participant characteristics (BMI, sex, education, and age) and allocated intervention group were related to dropout (dropout, no = 0, yes = 1) during the study. Repeated measures analyses were performed, using a general linear mixed model with a random intercept, to study changes during the study period (‘time’) and differences in changes between the intervention groups (‘group’, GI = 0 vs TI = 1) for the main outcome measures. These measures were objectively measured BMI, skin fold thickness and waist circumference, and self-reported PA and DI (intake of fat, snacks and sweetened drinks) (‘group*time’ interaction). This procedure allows for inclusion of cases with missing data, without replacement of missing values, and therefore includes all randomized participants. The ‘Type III Wald test’ was used to test overall statistical significance of the effects. The significance level (*P*) was set at .05 and tests were two-sided. All analyses were performed using SPSS 17.

## Results

### Participant Characteristics

**Figure 1:**
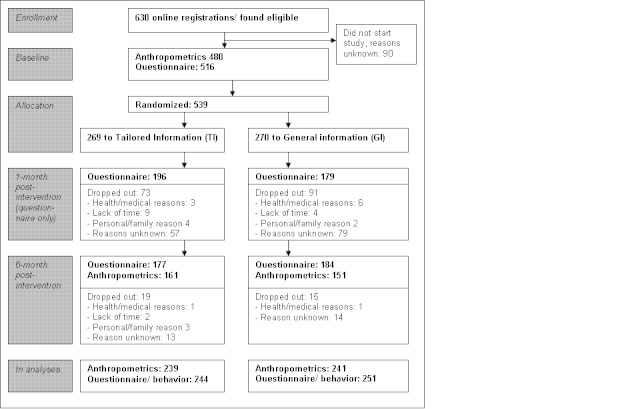
Participant flow for the GRIPP study.

In total, 630 people completed the online registration, and 539 enrolled in the study by completing the baseline questionnaire and/or anthropometric measures (Figure 1). The mean age of the participants was 47.8 years (SD 9.4), 31% were male and 11% had a low level of education. The mean BMI was 28.04 (SD 1.94). No baseline differences in socio-demographic characteristics, behavior and anthropometrics were observed between the intervention groups at baseline (*P*<.05, t-tests) (Table 1).

**Table 1:** Participant characteristics at baseline.

Group characteristics		Total (N = 539)	TI assigned group (n = 269)	GI assigned group (n = 270)
**Age, mean (SD)**		47.8 (9.4)	47.7 (9.2)	47.9 (9.7)
**Male sex, % (n)**		30.9 (164)	31.3 (84)	30.5 (80)
**Education, % (n)**				
	low	10.7 (49)	10.3 (24)	11.0 (25)
	medium	50.2 (231)	48.7 (113)	52.0 (118)
	high	39.1 (179)	40.9 (95)	37 (84)
**Anthropometric measures^a^**				
**BMI**	Mean kg/m^2^ (SD)	28.04 (1.94)	28.17 (2.02)	27.91 (1.85)
	% Normal weight (<25) (n)	4.2 (20)	4.2 (10)	4.1 (10)
	% Overweight (25-30) (n)	78.5 (377)	75.3 (180)	81.7 (197)
	% Obese (>30) (n)	17.3 (83)	20.5 (49)	14.1 (34)
**Waist circumference, mean centimeters (SD)**		95.78 (8.79)	95.89 (9.05)	95.66 (8.55)
**Skinfold thickness, mean centimeters (SD)**		8.82 (1.97)	8.87 (1.92)	8.77 (2.0)
**Self-reported behavior^b^**				
**Physical activity, mean minutes/day (SD)**		67.12 (54.3)	63.1 (50.4)	69.6 (42.4)
**Fat intake, mean points/week (SD)**		17.14 (6.1)	17.0 (6.0)	17.3 (6.2)
**Sweetened drinks, mean servings/day (SD)**		0.95 (1.2)	0.96 (1.2)	0.93 (1.2)
**Snacks, mean number/day (SD)**		2.28 (1.9)	2.2 (2.0)	2.3 (1.9)

^a^ N(total) = 480, n(TI group) = 239, n(GI group = 241)

^b^ N(total) = 457, n(TI group) = 231, n(GI group = 226)

### Loss to Follow-up

At baseline, 480 participants had their anthropometrics measured and 313 people at 6-month post-intervention (dropout 34.8%). Younger people were more likely to dropout between the two moments of anthropometric measures (OR age = 0.97, 95% CI 0.95-0.99). A total of 375 participants filled out the 1-month post-intervention (dropout 31%) and 361 people the 6-month post-intervention questionnaire (dropout 33%). Dropout between baseline and 1-month post-intervention questionnaires was more likely among men (OR sex = 0.56, 95% CI 0.35-0.89). Dropout between baseline and 6-month post-intervention was more likely among younger participants (OR age=0.97, 95% CI 0.95-0.99). No other differences were observed among those who completed the study and those who were lost after the first or second measurement.

### Intervention Effects

Repeated measures analyses showed that BMI did not change significantly over time (*P*=.09) (Table 2 and Table 3) and that there was no difference between the two groups (interaction effect group*time *P*=.09). Skin fold thickness increased significantly over time (*P*<.001) but there were no differences in change over time between the TI and GI group (interaction effect group*time *P*=.95). There was a statistically significant decrease in waist circumference over time (*P*<.001), but the change in time was not different between the study groups (interaction effect group*time *P*=.12).

**Table 2:** Crude means and difference of objectively measured anthropometric outcomes at baseline and 6-month post-intervention (N=480).

Outcome	Baseline mean (SD)		6-month post-intervention mean (SD)		Difference T0-T2	
	TI (n = 239)	GI (n = 241)	TI (n = 151)	GI (n = 161)	TI (n = 151)	GI (n = 161)
BMI, kg/m^2^	28.17 (2.02)	27.91 (1.85)	28.09 (2.36)	27.61 (2.03)	-0.08	-0.30
WC^a^, cm	95.89 (9.05)	95.66 (8.55)	94.41 (10.23)	93.20 (8.61)	-1.48	-2.46
SFT^a^, cm	8.87 (1.92)	8.77 (2.0)	9.67 (2.14)	9.47 (2.14)	+0.80	+0.70

^a^ WC = waist circumference, SFT = skin fold thickness

**Table 3:** Results of general linear mixed model analyses for objectively measured anthropometric outcome measures: Estimated marginal means and P values of time and group*time effects (N=480).

Outcome	Baseline estimated means		6-month post-intervention: estimated means		Type III tests *P* values	
	TI	GI	TI	GI	Time	Group*time
BMI, kg/m^2^	28.19	27.89	29.19	27.65	.09	.09
WC^a^, cm	95.87	95.66	94.62	93.48	<.001	.12
SFT^a^, cm	8.87	8.77	9.55	9.66	<.001	.95

^a^ WC = waist circumference, SFT = skin fold thickness

The time spent on physical activity decreased significantly in the total population (*P*=.002), but the change was not significantly different among the TI and GI (interaction effect group*time *P*=.44) (Table 4 and Table 5). Mean fat intake decreased statistically significantly between baseline measurement and post-intervention measurements (*P*<.001), but the decrease was similar in the two conditions (interaction effect group*time *P*=.74). Intake of sweetened drinks and snacks showed a similar pattern: intake decreased over time (sweetened drinks *P*<.001, snacks *P*<.001), but changes were not different between the intervention groups (interaction effect group*time: sweetened drinks *P*=.55, and snack intake *P*=.78).

Some of the behavioral outcomes measures (physical activity and intake of sweetened drinks and snacks) had a very skewed distribution. Log-transformation of these outcomes did not improve the fitted model and was not of influence on the time and intervention effects.

**Table 4:** Crude means and difference of specific self-reported behavior (DI and PA) at baseline, and 1- and 6-month post-intervention (N=495).

Outcome	Baseline mean (SD)		1-month post-intervention mean (SD)		6-month post-intervention mean (SD)		Mean difference T0-T1, T0-T2	
	TI (n = 231)	GI (n = 226)	TI (n = 196)	GI (n = 179)	TI (n = 177)	GI (n = 184)	TI	GI
Minutes PA/day	63.1 (50.4)	69.6 (42.4)	61.9 (56.5)	68.9 (51.8)	63.3 (53.6)	78.7 (60.7)	-1.2, +0.2	-0.7, +15.6
Fat intake/ week	17.0 (6.0)	17.3 (6.2)	15.3 (6.3)	15.7 (6.2)	15.4 (6.0)	15.9 (6.4)	-1.7, -1.6	-1.6, -1.4
Servings sweetened drinks/day	0.96 (1.2)	0.93 (1.2)	0.8 (1.3)	0.7 (1.2)	0.8 (1.1)	0.7 (1.0)	-0.16, 0.16	-0.23, -0.23
Snacks/ day	2.2 (2.0)	2.3 (1.9)	1.7 (1.6)	1.8 (1.6)	1.8 (1.4)	1.9 (1.5)	-0.5, -0.4	-0.5, -0.4

**Table 5:** Results of general linear mixed model analyses for specific self-reported behavior (DI and PA) at baseline, and 1- and 6-month post-intervention: Estimated marginal means and P values of time and group*time effects (N=495).

Outcome	Baseline estimated means		1-month post-intervention: estimated means		6-month post-intervention: estimated means		Type III tests *P* values	
	TI (n = 231)	GI (n = 226)	TI (n = 196)	GI (n = 179)	TI (n = 177)	GI (n = 184)	Time	Group*time
Minutes PA/day	63.06	69.58	61.94	68.90	63.25	78.71	<.001	.44
Fat intake/ week	17.94	16.94	15.47	15.89	15.23	15.99	<.001	.74
Servings sweetened drinks/day	0.96	0.94	0.82	0.74	0.81	0.67	<.001	.55
Snacks/ day	2.21	2.29	1.73	1.91	1.80	1.90	<.001	.78

### Process Evaluation

**Figure 2:**
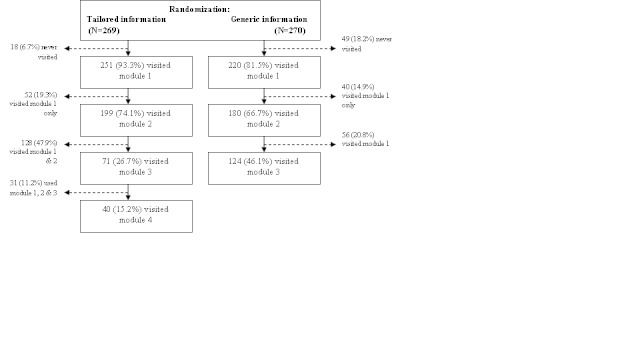
Attrition flowchart: server registration of use of general information intervention and tailored information intervention.

The login data showed that more than 80% (n = 272) of the respondents used the first module of their allocated intervention (Figure 2). About 15% (n = 41) completed four modules of the TI intervention and 46% (n = 124) completed the three modules of the GI intervention.

*T*-tests (Table 6) showed that participants in the TI group reported to have read statistically significantly less of the presented information (TI 4.07 vs GI 4.5, *P*<.001) and were slightly less positive about the lengths of the texts (TI 3.20 vs GI 3.07, *P =* .01), compared to the TI group. The information in the TI group was experienced as more ‘new’ (TI 3.11 vs GI 2.73, *P*=.003) and individualized (TI 3.20 vs GI 2.83, *P*=.001), compared with the GI. No differences were found for the usefulness (TI 3.48 vs GI 3.44) and attractiveness (TI 3.22 vs GI 3.16) of the information, the usefulness of the program as a tool for WGP (TI 2.90 vs GI 2.75), recommending it to others (TI 2.14 vs GI 2.14), and the overall grade (TI 6.6 vs GI 6.6).

**Table 6:** Process measures; means (SD) of appreciation of general information intervention and tailored information intervention, self-reported at 1-month post intervention, and t tests for differences between these groups.

Self-reported process measures^a^	Scale	TI mean (SD) (n = 162)	GI mean (SD) (n = 154)	*P* values
Amount of information read	Nothing (1)-All (5)	4.07 (1.0)	4.5 (0.8)	<.001
Length of the texts	Much too short (1)-Much too long (5)	3.20 (0.5)	3.07 (0.4)	.01
Information meant for me personally	Not at all (1)-Very much (5)	3.20 (1.0)	2.83 (1.0)	.001
New information	Totally disagree (1)-Totally agree (5)	3.11 (1.1)	2.73 (1.1)	.003
Useful information	Totally disagree (1)-Totally agree (5)	3.48 (0.9)	3.44 (0.9)	.68
Attractive design	Totally disagree (1)-Totally agree (5)	3.22 (0.9)	3.16 (0.9)	.58
Good tool for WGP	Totally disagree (1)-Totally agree (5)	2.90 (1.1)	2.75 (1.0)	.20
Recommend to others	No (1)-Maybe (2)-Yes (3)	2.14 (0.7)	2.14 (0.8)	.93
Overall grade	Very low (0)-Very high (10)	6.6 (1.4)	6.6 (1.3)	.69

^a^ Self-reported at 1-month post-intervention questionnaire, must have used at least the first module

## Discussion

In the present study we evaluated the effects of a carefully developed multi-session computer-tailored weight management intervention for overweight adults that was based on self-regulation theory and contained a number of self-regulation strategies. The results of the study showed that weight remained stable over time and that waist circumference and dietary behaviors slightly improved over six months, but that these improvements were not different from those in the generic information control group. Thus, even though some of the effects of the intervention were in the desired direction, in the present study we could not demonstrate that the elaborate intervention was more effective in inducing weight gain prevention than more basic generic information about weight management.

### Interpretation of the Results: Theory and Previous Evidence

Our intervention was solidly based on theory and evidence relevant for inducing weight maintenance and long-term changes in dietary intake and physical activity, and used the promising method of computer tailoring as an educational technique. It therefore had, in theory, good prospects for effects. The technique of computer tailoring has successfully been applied in interventions aimed at dietary intake, physical activity and weight loss [22,34,35] but no meta-analytic results are known for prevention of weight gain.

Our tailored intervention had a number of characteristics that have been indentified as having a positive influence on intervention efficacy [22,35], such as including 4 modules (‘contact moments’), and at least six behavior change strategies. Thus, the intervention had, in theory, good prospects to be effective.

Furthermore, based on the limited evidence with respect to effectiveness of interventions aimed at the prevention of weight gain [17], the results of our study fit in the pattern of mixed effects that have been found. One study found positive effects of an online weight management intervention [36], but this intervention aimed at a restriction of calorie intake to 1200-1500 calories/day, while our study aimed at a reduction of 100 kcal/day. It may be argued that a change of 100 kcal/day is too small to prevent weight gain or induce modest weight loss, but there is compelling evidence that this approach can in fact result in weight maintenance and modest weight loss, including for people who are overweight [37-39]. Our results are, furthermore, comparable to a number of other studies where the intervention group showed small or no effects on weight, compared to their control group [13,40,41].

The results of our study compare unfavorably to the results from 2 previous intervention studies conducted in the Netherlands [14,15]. These studies found effects on waist circumference and skin fold thickness [15] or waist circumference and weight [14], but had employees of companies as their target population and were more extensive in that it included email counseling [14] and changes in the environment [15]. Despite the planned theory and evidence-based development, the number of contacts, and the dynamic tailoring, our tailored intervention did not appear to be more effective than general information. Possible explanations may be the integration of self-regulation theory in an online intervention, methodological issues, and limited exposure to the intervention.

### Theoretical Basis

We used self-regulation theory as the main theoretical basis for the intervention. This theory is particularly suitable for management of chronic diseases, and behaviors and outcomes that need life long regulation, which is also the case for weight management. Self-regulation theory has been applied successfully in interventions aimed at weight-loss among young adults, asthma management, and diabetes [42-44]. Many successful interventions that were based on self-regulation have been intensive counselor-led interventions, in which participants are guided through all the important steps of self-regulation (monitoring, goal setting, action planning, evaluation, and adaptation). However, in the present study we had to apply the self-regulation principles and strategies in a computerized program that had to guide the participants through all the steps of self-regulation. Although feasible, incorporating principles from self-regulation theory and self-regulation strategies in an online self-management intervention may not translate into the same results as when implemented in a face-to-face counseling session. That is, there are fewer possibilities for instructions for use, a smaller variety of options, and use of the intervention components may be less optimal, both in quality and frequency of use. There is indeed evidence that, for example, implementation intentions have been of better quality when developed in the presence of a researcher who reviewed the plans, compared to plans that were developed without a counselor present [45]. Goals and plans that lack quality have previously been reported from unguided interventions [46,47].

### Exposure to the Intervention

Even though multiple visits are associated with higher efficacy of (computer-tailored) interventions [48], it has also been well documented that attrition is high in online interventions with multiple sessions [49-51]. To improve the likelihood that participants would revisit the intervention, we incorporated some of the elements that had been indicated as potentially associated with more revisits in previous research [52] in the intervention. In follow-up visits participants could monitor progress, could access new elements of the intervention, could review recipes and tips on the website that were refreshed every 2 weeks and participants received email reminders to visit the website every 2 weeks. Nevertheless, logon rates decreased over time. As a consequence, large numbers of participants in the intervention group were not exposed to parts of the intervention in which important self-regulation strategies, such as monitoring, feedback, and coping planning were incorporated. Therefore, the ‘dynamic’ tailoring (eg, feedback on progress), which is one element of computer tailoring that can increase effect size [22,53], was not delivered to large numbers of participants. Similar (low) use of self-regulation components has been observed before among a study sample of overweight adults [54].

Possible reasons for dropout were the number and length of the tailored modules, and perhaps the ineffectiveness of the reminders. In order to increase use and effectiveness, we should improve these aspects, for example, by using other media such as short message service (SMS) as reminders [55]. Direct contact, in order to motivate people and to teach the steps of self-regulation and problem solving skills [48], may be useful to increase involvement. Furthermore, although the tailored intervention was appreciated a little bit more than the general information intervention, the overall appreciation was just sufficient. Improving the intervention on these aspects may have a positive influence on use and effect.

### Methodological Considerations

Kremers et al [17] found in their review that interventions aimed at weight management have small effect sizes (mean effect size of 0.06). This means that studies need to be powered to detect such small effect sizes. Our study was sufficiently powered to detect a difference of 0.4 BMI points, based on the assumption that weight would remain stable or be slightly reduced in the intervention group, and that weight would show the usual annual increase of 0.6 kg among the control group. However, weight did not increase in the control group in the actual study, but remained stable. Even though initially our sample size was sufficient for detecting the anticipated effect, retention of participants in the study was much lower than we had anticipated, thus reducing the actual power of the study. However, the size of the effect appears to be very small, indicating that lack of power was not the most important reason for not detecting an effect. Rather, the effect in the control group, whether it was caused by participation in the study, completion of the questionnaire, or the exposure to generic information, was larger than expected. The high dropout from the study is not unusual [56], but is a serious concern, and at this moment we do not know the reasons for this high dropout.

It is possible that we could have detected an effect had we compared the intervention with a no-intervention control group. There is evidence that the effect size of a computer-tailored intervention depends on the comparison condition: comparing a tailored intervention to a generic message (*r* = 0.058) results in smaller effect sizes than comparing it to a no-treatment group (*r* = 0.11) [35]. However, comparing the intervention to a no information control group can only provide insight into the effects of the intervention per se and cannot provide insight in the effectiveness of a specific technique, such as tailoring or self-regulation. In the present study we were interested in examining the effects in comparison to a control group that received ‘usual care’ in the form of generic information about weight management that can also be found on the website of the Dutch Nutrition Bureau. The technique of computer-tailoring and the incorporation of self-regulation strategies as incorporated and implemented in the present study appeared no more effective than the already available information on Dutch websites.

Another methodological issue that is worth discussing is the effect of offering respondents the option to choose their own behavioral change. Our second hypothesis concerned differences in dietary and physical activity behaviors. Changes in dietary and PA behaviors may precede or mediate changes in anthropometric outcome measures and are therefore relevant to study. Even though there was a time effect for some of these behaviors, there were no differences between the intervention and control group.

Lack of effects on the behavioral outcome measures was somewhat expected since participants could choose from 10 changes in DI and PA (eg, increase active transport with 20 minutes per day), and even more options within these subgroups (eg, cycling or walking), based on their current dietary and PA behavior. This results in small groups of people who chose for the same change to make, which makes it difficult to detect differences between the study groups in the dietary and PA sub-behaviors. Limited power for detection of differences in relevant underlying behaviors is one of the consequences of dynamic and personally adapted interventions.

### Strengths and Limitations

Strengths of the study are its randomized controlled design and the use of objective measures of BMI, waist circumference and skin fold thickness. Limitations are the self-reported measures for physical activity and dietary intake, which may have resulted in less reliable outcomes. The baseline level of PA was high among all participants in the study, which may be due to over-reporting. If the participants also over-reported their PA level in the tailored intervention, this may have resulted in less room for improvement, limiting the potential intervention effect. Another limitation is that participants in the intervention and control condition were not exposed to an equal number of program sessions. However, we do not expect that this had a large effect on the study outcomes, since the tailored intervention (with more elaborate content) was not more effective than the generic information. Furthermore, we had a 6-month assessment as the longest follow-up. We cannot rule out that differences between the study groups would become more apparent after a longer follow-up period. Although dropout was high, it was similar in both conditions and mainly related to age, as in other studies [49-51]. However, due to the high dropout the results cannot be generalized to other populations than those who remained participating in the study.

### Conclusion

The carefully developed online, computer-tailored weight management intervention for adults who are overweight resulted in stable weight, and changes in dietary intake in the desired direction, but the tailored intervention was not more effective than generic information. A possible reason for the absence of intervention effects is sub-optimal use of the intervention, and the self-regulation components. Further research is therefore needed to gain more insight into how the intervention and exposure to its contents can be improved.

## Multimedia Appendix 1

CONSORT-EHEALTH checklist V1.6 [57].

## Abbreviations

BMI: Body Mass Index
DI: Dietary intake
GI: Generic information intervention
PA: Physical activity
SFT: Skin fold thickness
SMS: Short message service
TI: Tailored intervention
WC: Waist circumference
